# Comprehensive evaluation of ACMG/AMP-based variant classification tools

**DOI:** 10.1093/bioinformatics/btaf623

**Published:** 2026-02-13

**Authors:** Tohid Ghasemnejad, Yuheng Liang, Khadijeh Hoda Jahanian, Milad Eidi, Arash Salmaninejad, Seyedeh Sedigheh Abedini, Fabrizzio Horta, Nigel H Lovell, Thantrira Porntaveetus, Mark Grosser, Mahmoud Aarabi, Hamid Alinejad-Rokny

**Affiliations:** UNSW BioMedical Machine Learning Lab (BML), School of Biomedical Engineering, UNSW Sydney, Sydney, NSW 2052, Australia; UNSW BioMedical Machine Learning Lab (BML), School of Biomedical Engineering, UNSW Sydney, Sydney, NSW 2052, Australia; Center of Excellence in Precision Medicine and Digital Health, Department of Physiology, Faculty of Dentistry, Chulalongkorn University, Bangkok 10330, Thailand; The International ImMunoGeneTics Information System (IMGT), National Center for Scientific Research (CNRS), Institute of Human Genetics (IGH), University of Montpellier (UM), Montpellier 34396, France; Center for Individualized Medicine, Mayo Clinic, Rochester, MN 55905, United States; UNSW BioMedical Machine Learning Lab (BML), School of Biomedical Engineering, UNSW Sydney, Sydney, NSW 2052, Australia; Fertility & Research Centre, Discipline of Women’s health, School of Clinical Medicine and the Royal Hospital for Women, University of New South Wales, Sydney, NSW 2031, Australia; Dept O&G, Monash University, Melbourne, VIC 3168, Australia; City Fertility, Research Support Unit, Sydney, NSW 2000, Australia; School of Biomedical Engineering, UNSW Sydney, Sydney, NSW 2052, Australia; Center of Excellence in Precision Medicine and Digital Health, Department of Physiology, Faculty of Dentistry, Chulalongkorn University, Bangkok 10330, Thailand; Unit 82/26-32, 23Strands, Pirrama Rd, Pyrmont, NSW 2009, Australia; Departments of Pathology, and Obstetrics, Gynecology and Reproductive Sciences, University of Pittsburgh School of Medicine, Pittsburgh, PA 15213, United States; UNSW BioMedical Machine Learning Lab (BML), School of Biomedical Engineering, UNSW Sydney, Sydney, NSW 2052, Australia; Visiting Scholar (Collaborative Projects), Center of Excellence in Precision Medicine and Digital Health, Chulalongkorn University, Bangkok 10330, Thailand

## Abstract

**Motivation:**

The American College of Medical Genetics and Genomics/Association for Molecular Pathology (ACMG/AMP) guidelines represent the gold standard for clinical variant interpretation. Despite the widespread adoption of ACMG/AMP guidelines, a comprehensive comparison of the software tools designed to implement them has been lacking. This represents a significant gap, as clinicians require evidence-based guidance on which tools to use in their practice.

**Results:**

We benchmarked four ACMG/AMP-based tools (Franklin, InterVar, TAPES, Genebe) selected from 22 tools, and compared their performance with LIRICAL, a top-performing phenotype-driven tool, using 151 expert-curated datasets from Mendelian disorders. Selection criteria included free availability, VCF compatibility, operational reliability, and not being disease-specific. Our evaluation framework assessed top-*N* accuracy (*N* = 1, 5, 10, 20, 50), retention rates, precision, recall, *F*1 scores, and area under the curve (AUC). Statistical validation employed bootstrap confidence intervals (*n* = 1000) and Friedman tests. LIRICAL (68.21%) and Franklin (61.59%) demonstrated superior top-10 variant prioritization accuracy in Mendelian disorders, significantly outperforming other tools (*P* = .0000). Results demonstrate that tools with advanced phenotypic integration significantly outperform those relying primarily on genomic features.

**Availability and implementation:**

All data and source code required to reproduce the findings of this study are openly available in the Code Ocean repository at https://doi.org/10.24433/CO.6562438.v1.

## Graphical Abstract

**Graphical Abstract:**
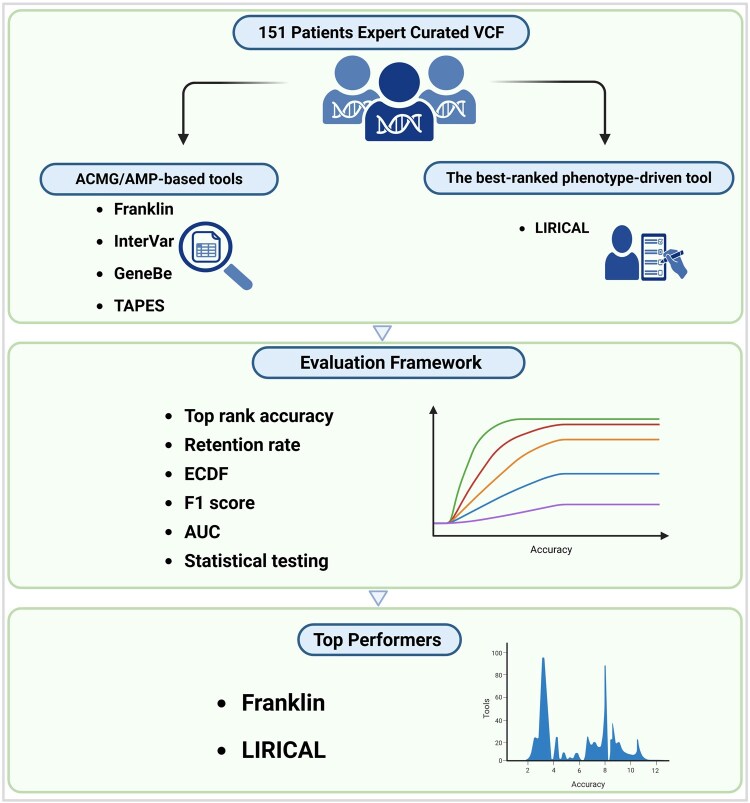

## 1 Introduction

**Figure 1.**
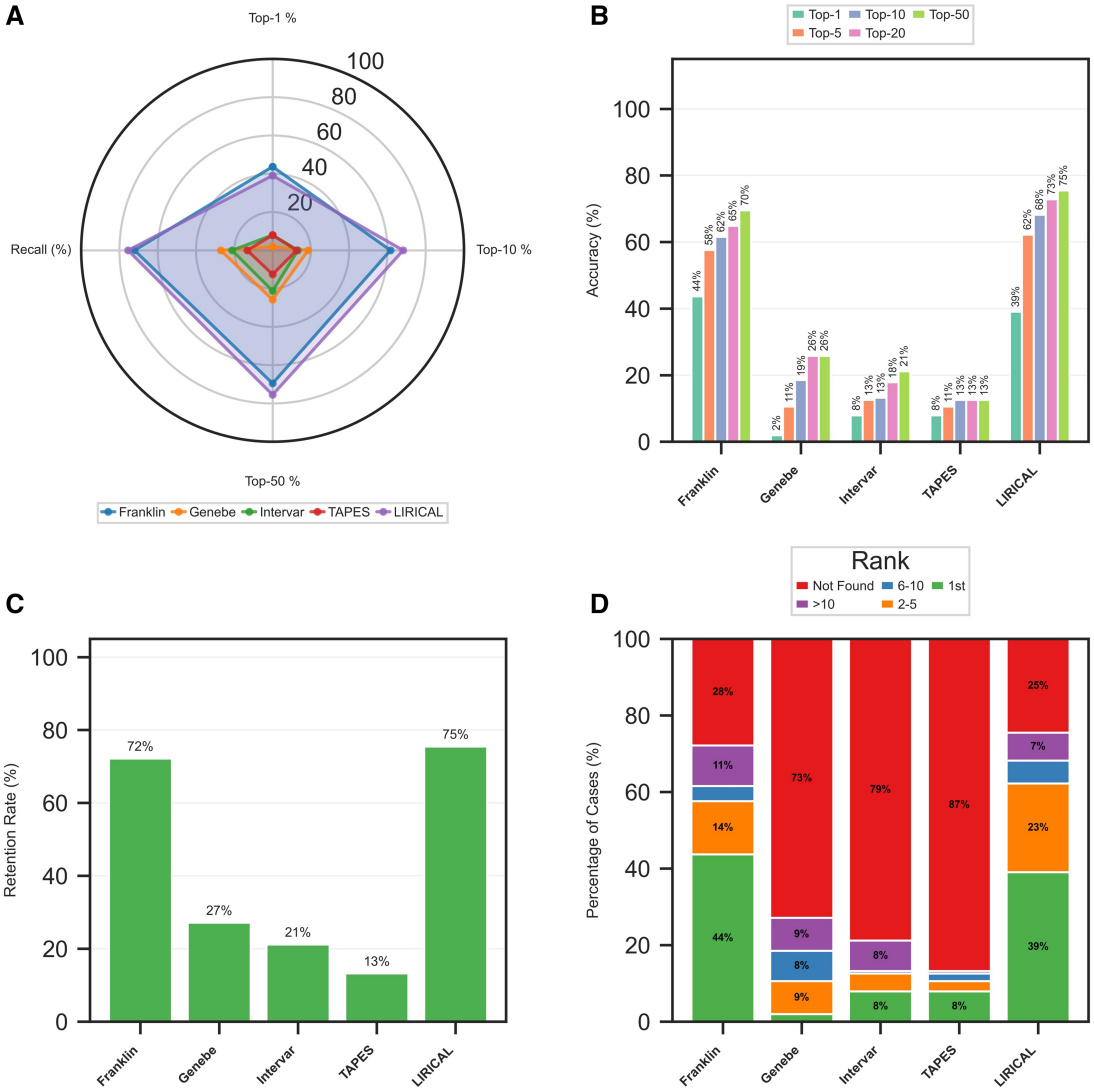
Overview of variant prioritization performance. (A) Radar plot comparing tool performance across key accuracy and recall metrics. (B) Bar chart detailing the top-*N* accuracy at various thresholds. (C) Gene retention rates for each tool. (D) Distribution of causative gene ranks across different tiers.

The emergence of next-generation sequencing (NGS) has revolutionized traditional diagnostic methods, representing a significant technological advancement (Koboldt 2020). As the technical capabilities of NGS-based tests advance at a rapid pace, it becomes essential to establish the clinical effectiveness and utility of these methods across diverse communities. This relies heavily on the ability to thoroughly, reproducibly, and timely interpret sequence variants in a clinical setting (Chen *et al.* 2015). Evaluating the pathogenicity of genetic variants is a complex task that depends on various sources of information. These dynamic, frequently updated sources lead to variations in interpretation across different laboratories (Garber *et al.* 2016). Various computational tools have been developed for interpreting variants. These tools can be broadly categorized based on two primary objectives: *in silico* tools that predict the harmful effects of a variant on a gene product (e.g. protein, splice site) (Mahmood *et al.* 2017) and phenotype-driven tools that identify disease-causing variants in patients based on phenotypic features (Kelly *et al.* 2022). The use of different approaches has led to the challenge of conflicting pathogenicity assessments for variants (Kumar 2007). To address this challenge, the American College of Medical Genetics and Genomics (ACMG) and the Association for Molecular Pathology (AMP) established guidelines to promote standardization in variant interpretation (Richards *et al.* 2015). These guidelines, summarized in Table 1, available as supplementary data at *Bioinformatics* online and Fig. 1, available as supplementary data at *Bioinformatics* online, introduced a scoring system that uses various information such as functional data, population frequency, and *in silico* predictions to classify variants as pathogenic, likely pathogenic, variant of uncertain significance (VUS), likely benign, and benign (Richards *et al.* 2015).

Considering the widespread adoption of the ACMG/AMP guidelines for classifying variants in genes with Mendelian inheritance patterns (Niehaus *et al.* 2019), automated tools have been developed for quick and consistent variant classification (Nicora *et al.* 2018, 2022, Kopanos *et al.* 2019, Xavier *et al.* 2019, Li *et al.* 2021, Munté *et al.* 2023). These tools can take the form of pipelines (Scott *et al.* 2019, Xavier *et al.* 2019, Munté *et al.* 2023) or web platforms (Franklin by Genoox 2025; Whiffin *et al.* 2018; Kopanos *et al.* 2019) that implement the guidelines directly or transform them into a probabilistic framework. Furthermore, machine learning (ML) approaches have utilized data-driven methods to distinguish between pathogenic and benign variants (Nicora *et al.* 2022).

In contrast to tools that follow the ACMG/AMP guidelines, phenotype-driven tools have emerged that assign high weight to patient phenotypes, enabling them to filter through thousands of variants by identifying those associated with diseases that best match the patient’s specific clinical manifestations, thereby significantly reducing the interpretive burden on clinicians (Robinson *et al.* 2020). While previous research has evaluated *in silico* predictors (Mahmood *et al.* 2017, Cannon *et al.* 2023) and phenotype-driven approaches (Kelly *et al.* 2022, Yuan *et al.* 2022), no comprehensive comparison of tools that follow the ACMG/AMP guidelines has been conducted. In this study, using expert-curated diagnoses from VCF files, our objectives were to: (i) benchmark the performance of ACMG/AMP-based tools, (ii) compare their effectiveness against LIRICAL (Robinson *et al.* 2020), a leading phenotype-driven tool with established excellence in prior benchmarking studies (Kelly *et al.* 2022, Yuan *et al.* 2022), and (iii) provide evidence-based recommendations for clinical tool selection. This analysis provides critical insights for improving automated variant interpretation platforms and informing the development of hybrid approaches.

## 2 Materials and methods

### 2.1 Study design and tool selection

We developed a comprehensive and systematic search strategy (Supplementary file, available as supplementary data at *Bioinformatics* online) to identify relevant studies and automated variant classification tools that implement ACMG/AMP guidelines. The search encompassed PubMed databases and included manual screening of ACMG/AMP-compliant tools referenced in identified publications. Search terms combined keywords related to ACMG/AMP guidelines, automated variant interpretation, and computational pathogenicity prediction. Studies and tools were systematically evaluated and included based on four key criteria: (i) focus on automated tools capable of processing Variant Call Format (VCF) files, (ii) free availability through open-source platforms or unrestricted access, (iii) demonstrated reliable operation without significant technical errors during implementation, and (iv) not restricted to specific diseases or conditions.

### 2.2 Dataset and ethical considerations

This study leveraged a de-identified dataset comprising 151 VCF files from patients with Mendelian disorders. These were sourced through collaborations with clinical diagnostic laboratories (see Table 2, available as supplementary data at *Bioinformatics* online, for details). All samples were obtained with written informed consent from participants or their legal guardians for research purposes, under protocols approved by the Chulalongkorn University Institutional Review Board (No. 264/62). The research was conducted in accordance with the Declaration of Helsinki, and all patient identifiers were removed prior to data transfer, ensuring adherence to institutional data protection policies. The benchmark dataset was built through multiple curation steps, with two independent clinical geneticists evaluating each VCF file according to ACMG/AMP guidelines. All cases were processed using a standardized pipeline, ensuring variant normalization and nomenclature harmonization per Human Genome Organization (HUGO) Gene Nomenclature Committee (HGNC) and Human Genome Variation Society (HGVS) guidelines.

### 2.3 Phenotypic data standardization

For tools requiring phenotypic input (e.g. LIRICAL, Franklin), standardized Human Phenotype Ontology (HPO) terms were derived from clinical records. This involved extracting symptoms, clinical features, and phenotypic abnormalities, mapping them to HPO terms using ontology tools (https://doc2hpo.wglab.org/), and validating selections through clinical review. This approach ensured uniform phenotypic data representation across all tools evaluated.

### 2.4 Variant prioritization and tool-specific processing

Each tool employed unique variant prioritization methods, necessitating tailored data processing. For ACMG/AMP-based tools, outputs were sorted from pathogenic to benign using a unified scoring system (pathogenic = 5, likely pathogenic = 4, VUS = 3, likely benign = 2, benign = 1) to standardize classifications. LIRICAL, utilizing a probabilistic likelihood ratio model, was processed using its native rank outputs directly, preserving its explicit ranking structure.

### 2.5 Analytical framework

To provide a holistic evaluation of tool performance, we employed a multi-faceted analytical framework. The assessment began with fundamental accuracy metrics, including Top-*N* accuracy at clinically relevant thresholds (*N* = 1, 5, 10, 20, 50). We then evaluated each tool’s filtering efficiency through its retention rate, which measures the ability to include the causative gene, alongside its corresponding filtered-out rate. To understand the tradeoffs between specificity and sensitivity, we analyzed precision, recall, and *F*1 score, which collectively measure a tool’s ability to correctly identify causative variants (precision) while ensuring no true causative variants are missed (recall).

We visualized overall ranking behavior using Empirical Cumulative Distribution Functions (ECDFs) and quantified it through the area under the receiver operating characteristic curve (AUC), which penalizes tools for incorrectly filtering out true variants. We synthesized performance across key dimensions using radar plots for intuitive multi-metric comparison. Finally, we established statistical significance using bootstrap confidence intervals (*n* = 1000) and the non-parametric Friedman test to ensure robust comparisons across the toolset.

### 2.6 Performance metrics

We assessed tool performance using gene prioritization metrics. Top-*N* accuracy represented the proportion of cases where the causative gene appeared within the top-*N*-ranked genes. This metric directly measures a tool’s ability to place the correct gene near the top of its results list, reflecting its immediate clinical utility. This single metric was used for all rank thresholds (e.g. *N* = 1 for exact match rate) and was calculated using Equation (1):


(1) $$\text{Top}-N\;a\text{ccuracy}=\frac{N_{\text{rank}\leq N}}{N_{\text{total}}}\times 100$$

where $N_{\text{rank}\leq N}$ represents cases with the causative gene ranked within the top (*N*) positions, and $N_{\text{total}}$ is the total number of cases analyzed. The retention rate, calculated using Equation (2), measures the proportion of cases where the causative gene was successfully included in the tool’s first 100 outputs.


(2) $$\text{Retention}\;r\text{ate}=\frac{N_{\text{found}}}{N_{\text{total}}}\times 100$$

where $N_{\text{found}}$ represents cases where the causative gene was found in the tool’s output. The filtered-out rate, quantified as the proportion of cases where the causative gene was completely absent from the tool’s output in the specific threshold, was calculated through Equation (3):


(3) $$\text{Filtered}-o\text{ut}\;r\text{ate}=\frac{N_{\text{not}\;\text{found}}}{N_{\text{total}}}\times 100$$

where $N_{\text{not}\;\text{found}}$ represents cases where the causative gene was not found. Having established these core performance metrics, we next analyzed the distribution of causative genes across ranking positions.

### 2.7 Rank distribution analysis

To evaluate clinical utility, we categorized gene ranks into groups reflecting typical review workflows: 1st (minimal effort), 2nd–5th (feasible review), 6th–10th (for complex cases), >10th (challenging for routine use), and FO (filtered-out). The aim of this analysis is to provide a granular view of where the correct gene typically falls in the rankings, which is a strong indicator of the real-world effort required to find it.

### 2.8 Comparative analysis

We employed several analytical approaches to compare prioritization accuracy across tools. We calculated precision at different rank thresholds (1, 5, 10, 20, 50) through Equation (4):


(4) $$\text{Precision}\;(k)=\frac{\sum_{i=1}^{N_{\text{total}}}1[\text{causative}\;{\text{gene}}_{i}\in \text{top}-k_{i}]}{\sum_{i=1}^{N_{\text{total}}}\min\left(k,|L_{i}|\right)}$$

where $N_{\text{total}}$ represents the total number of samples analyzed, $1[\text{causative}\;{\text{gene}}_{i}\in \text{top}-k_{i}]$ is an indicator function that equals 1 if the causative gene for sample i is found within the top k ranks (0 otherwise), ∣Li∣ represents the number of genes in the output for sample i, and $\min\left(k,|Li|)\right)$ ensures we only count actual ranked genes when a tool returns fewer than k genes. This formulation calculates precision as the proportion of true positives among all genes ranked at position k or higher across all samples, directly reflecting the clinical review burden. We also calculated recall. This metric measures the tool’s overall success rate in identifying the causative gene, irrespective of its rank. It was calculated through Equation (5):


(5) $$\text{Recall}=\frac{\text{TP}}{\text{TP}+\text{FN}}$$

where TP represents cases where the causative gene was found in the tool’s output, and FN represents cases where the causative gene was missing. We calculated the *F*1 score as the harmonic mean of precision and recall. This score provides a single, balanced measure of performance, rewarding tools that both successfully identify the causative gene and rank it first. Precision at rank threshold 1 was used in calculations to focus on the most clinically relevant ranking scenario, as shown in Equation (6):


(6) $$F_{1}=2\times \frac{\text{Precision}@1\times \text{Recall}}{\text{Precision}@1+\text{Recall}}$$

We created empirical cumulative distribution functions (ECDFs) of ranks to visualize overall ranking behavior. This visualization illustrates the probability of finding the causative gene at or before any given rank, offering a comprehensive overview of a tool’s ranking efficiency. It was calculated through Equation (7):


(7) $$F_{n}(x)=\frac{1}{n}\sum_{i=1}^{n}I({\text{rank}}_{i}\leq x)$$

We calculated the area under the receiver operating characteristic (ROC) curve (AUC) to evaluate each tool’s ability to discriminate between causative and non-causative genes. This single score summarizes a tool’s ability to consistently rank the true causative gene higher than non-causative genes, accounting for both ranking and filtering performance. It was calculated through Equation (8):


(8) $$\text{AUC}=\int_{0}^{1}\text{TPR}(t)\;d(\text{FPR}(t))$$

where TPR is true positive rate and FPR is false positive rate. Each gene received a score based on ranking position (higher ranks = higher scores). Causative genes served as the positive class, while all other genes served as the negative class. FO’s received a score of 0 to penalize excessive filtering. We also created a multidimensional performance visualization using radar plots to visualize performance characteristics across four key metrics: Top-1 accuracy (Equation (1) with *N* = 1), Top-5 accuracy (Equation (1) with *N* = 5), Top-10 accuracy (Equation (1) with *N* = 10), and overall accuracy (Equation (5), recall). These radar plots provide an intuitive visualization of tool performance across multiple dimensions simultaneously, allowing identification of tools with balanced strengths across several metrics rather than those excelling in only one aspect.

### 2.9 Statistical methods

To evaluate performance differences between the tools, we developed a statistical framework using complementary analyses. First, we calculated bootstrap confidence intervals (*n* = 1000) for our key metrics, which allowed us to estimate the reliability of our findings without assuming normal distributions. The bootstrap procedure is implemented using the following equation:


(9) $$95\%\;\text{CI}=[\theta_{0.025}^{*},\theta_{0.975}^{*}]$$

where $\theta_{\alpha /2}^{*}$ represents the *α*/2 quantile of the bootstrap distribution. For comparing multiple tools simultaneously, we employed the Friedman test, a non-parametric alternative to repeated measures ANOVA that avoids distributional assumptions through Equation (10):


(10) $$\chi_{r}^{2}=\frac{12}{nk(k+1)}\sum_{j=1}^{k}R_{j}^{2}-3n(k+1)$$

where n is the number of samples, k is the number of tools, and $R_{j}\;$is the sum of ranks for the *j* tool. These statistical methods were selected due to their suitability for non-parametric, matched datasets where the same variant classification tools were repeatedly evaluated across identical sets of variant cases. The Friedman test and bootstrap-derived confidence intervals provide robust comparative analysis without requiring assumptions about data distribution.

## 3 Results

### 3.1 Study design and tool selection

According to our search strategy (Supplementary file, available as supplementary data at *Bioinformatics* online), we identified 22 studies as relevant to automated variant classification based on the ACMG/AMP guidelines from an initial pool of 537 studies. The details of these 22 tools can be found in Table 1. Notably, some of these tools have universal application for variant classification, independent of the specific disease (Franklin by Genoox 2025; Li and Wang 2017, Kopanos *et al.* 2019, Scott *et al.* 2019, Xavier *et al.* 2019, Stawiński and Płoski 2024), while others are tailored for particular diseases, such as cardiovascular disease (Nicora *et al.* 2018, Whiffin *et al.* 2018), hearing loss (Peng *et al.* 2021, Melidis *et al.* 2022), cancer (Ravichandran *et al.* 2019, Li *et al.* 2021, Nakken *et al.* 2021, Karalidou *et al.* 2022, Munté *et al.* 2023), maturity-onset diabetes of the young (Liu *et al.* 2020), and mitochondrial missense variants (Bianco *et al.* 2023). We classified these tools into two groups based on their respective applicability: disease-independent tools and disease-specific tools. From an initial pool of 22 tools, we selected four tools that implement ACMG/AMP guidelines: Franklin (Franklin by Genoox 2025), Genebe (Stawiński and Płoski 2024), Tool for Assessment and Prioritisation in Exome Studies (TAPES) (Xavier *et al.* 2019), InterVar (Li and Wang 2017), based on inclusion criteria. We also included LIRICAL (Robinson *et al.* 2020) in our analysis, which takes a completely different approach by focusing primarily on phenotype-driven variant prioritization, and showed better performance in variant prioritization in recent benchmarking studies (Kelly *et al.* 2022, Yuan *et al.* 2022). By including tools with these different approaches, we could make meaningful comparisons between purely guideline-driven methods and those that incorporate phenotypic information across a wide range of genetic conditions.

**Table 1.** Characteristics and selection status of ACMG/AMP-based variant classification tools.

Tool type	Tool name	Analytical scope	VCF compatible	Open-source	Bulk analysis	Implemented ACMG criteria	Exclusion/inclusion status	Access	Ref
Disease-independent tools	InterVar	Universal	Yes	Yes	Yes	PVS1, PS1, PS4, PM1, PM2, PM4, PM5, PP2, PP3, PP5, BA1, BS2, BP1, BP3, BP4, BP6, BP7	Meets inclusion criteria	Web: https://wInterVar.wglab.org/ GitHub: https://github.com/WGLab/InterVar	Li and Wang (2017)
	Franklin	Universal	Yes	No	No	PVS1, PS1, PS4, PM1, PM2, PM4, PM5, PP2, PP4, PP3, PP5, BA1, BS2, BP1, BP3, BP4, BP6, BP7	Meets inclusion criteria	Web: https://franklin.genoox.com/clinical-db/home	Franklin by Genoox (2025)
	TAPES	Universal	Yes	Yes	Yes	BA1 BS1, BS2, BS3, BP1, BP3, BP4, BP6, BP7, PVS1, PS1, PS2, PS3, PS4, PM1, PM2, PM4, PM5, PP2, PP3, PP5	Meets inclusion criteria	GitHub: https://github.com/a-xavier/tapes	Xavier *et al.* (2019)
	VarSome	Universal	No	NA	Yes	PVS1, PS1, PS3, BS3, PM1, PM2, PM4, PM5, PP2, BP1, PP3, BP4, PP5, BP6, BA1, BS2, BP3, BP7	Requires paid subscription	Web: https://varsome.com/	Kopanos *et al.* (2019)
	ClinGen	Universal	No	No	No	NA	Incompatible with VCF file format	Web: https://curation.clinicalgenome.org/	Preston *et al.* (2022)
	eVai	Universal	Na	Na	NA	Please refer to Table 2 of the supplementary file of the reference	Requires paid subscription	Web: https://github.com/GiovannaNicora/MLVar	Nicora *et al.* (2022)
	MAGI-ACMG	Universal	No	No	No	PVS1, PS1, PS2, PS3, PS4, PM1, PM2, PM3, PM4, PM5, PM6, PP1, PP2, PP3, PP4, PP5, BA1, BS4, BP1, BP2, BP3, BP4, BP5, BP6, BP7, P_POT	Web platform execution failure	Web: http://magiacmg.magiclinici.it : 8805	Cristofoli *et al.* (2023)
	CharGer	Universal	Yes	Yes	Yes	PVS1, PS1, PSC1,^a^ PMC1,^b^ PM1, PM2, PM4, PM5, PP2, PP3, PPC1,^c^ PPC2,^d^ BP4, BMC1,^e^ BSC1,^f^ BA1	Universal benign classification bias	GitHub: https://github.com/ding-lab/	Scott *et al.* (2019)
	Varcard2	Universal	Yes	No	No	PVS1, PS1, PS2, PS3, PS4, PM1, PM2, PM4, PM5, PM6, PP1, PP2, PP3, BP1, BP3, BP4, BP7, BS1, BS2, BS3, BS4, BA1	Web platform execution failure	Web: https://www.genemed.tech/varcards2/#/index/home	Wang *et al.* (2024)
	Genebe	Universal	Yes	Yes	Yes	PVS1, PS1, PM1, PM2, PM4, PM5, PP2, PP3, PP5, BA1, BS1, BS2, BP1, BP3, BP4, BP6, BP7	Meets inclusion criteria	Web: https://genebe.net.	Stawiński and Płoski (2024)
Diseases-specific tools	MODY-RELATED GENES	Maturity-onset diabetes of the young	NA	No	NA	PVS1, PS1, PS4, PM1, PM2, PM4, PM5, PP2, PP3, PP5, BA1, BS1, BP4, BP6	Platform unavailability	NA	Liu *et al.* (2020)
	GenOtoScope	Hearing loss	Yes	Yes	Yes	PVS1, PS1, PM1, PM2, PM4, PM5, PP3, BA1, BS1, BP3, BP4, BP7	Pipeline execution failure	Web: http://genotoscope.mh-hannover.de : 5000 GitHub: https://github.com/damianosmel/GenOtoScope	Melidis *et al.* (2022)
	VIP-HL	Hearing loss	No	No	No	PVS1, PS1, PM1, PM2, PM4, PM5, PP3, BA1, BS1, BS2, BP3, BP4	Incompatible with VCF file format	Web: Nohttp://hearing.genetics.bgi.com	Peng *et al.* (2021)
	HTAADVar	Heritable thoracic aortic aneurysm and dissection	No	No	No	PVS1, PS1, PM5, PS2, PM6, PS3, BS3, PM2, BA1, BS1, PS4, BS2, PM1, PM4, BP3, PP2, PP1, BS4, PP3, BP4, BP7, PP4, BP2, BP5	Very specific utilization	Web: http://htaadvar.fwgenetics.org	Zhou *et al.* (2022)
	CardioVAI	Cardiovascular diseases	No	no	No	PVS1, PS1, PS3, PM1, PM2, PM4, PM5, PP2, PP3, PP5, BA1, BS1, BS3, BP1, BP3, BP4, BP6, BP7, BP8	Requires paid subscription	Web: https://cardiovai.engenome.com/	Nicora *et al.* (2018)
	CardioClassifier	Cardiovascular diseases	Yes	no	Yes	PVS1, PS1, PS4, PM1, PM2, PM4, PM5, PP2, PP3, BA1, BS1, BP3, BP4, BP7	Failed implementation due to system error	Web: https://www.cardioclassifier.org	Whiffin *et al.* (2018)
	MARGINAL	BRCA1/BRCA2 Genes	No	No	NA	PVS1, BA1, BS1, PM2, PP3, BP4. BP7 PP2, BP1, PM1, PM4, BP3, PS4, PS1, PM5	File size limitation (>50 MB not supported)	GitHub: https://github.com/vasilikikaral/MARGINAL-software	Karalidou *et al.* (2022)
	APOGEE 2	Mitochondrial variants	No	no	NA	NA	Platform unavailability	GitHub: https://github.com/mazzalab/playgrounds	Bianco *et al.* (2023)
	vaRHC	Cancer	Yes	Yes	Yes	PVS1, PS3, PM1, PM2, PM4, PM5, PP2, PP3, PP5, BA1, BS1, BS2, BS3, BS4, BP1, BP2, BP3, BP4, BP5, BP6, BP7	Limited variant processing capacity	GitHub: https://github.com/emunte/vaRHC	Munté *et al.* (2023)
	CPSR	Cancer	Yes	Yes	Yes	Please refer to Table 2 of the supplementary file of the reference	Specific for cancer	GitHub: https://github.com/sigven/cpsr	Nakken *et al.* (2021)
	Cancer SIGVAR	Cancer	Yes	Yes	Yes	PVS1, PS1, PM4, PM5, BP1, BP3, PP3, BA1, BS1, BS2, BS3, BP2, BP4, BP6 BP7	Web platform execution failure	Web: http://cancersigvar.bgi.com/	Li *et al.* (2021)
	PathoMan	Cancer	No	no	No	PVS1, PS1, PM5, PS3, BS3, PS4, PM2 BA1 BS1, BS2, PM1, PM4 BP3, PP3, BP4, PP5, BP6, BP7, PP2, BP1	Web platform execution failure	Web: https://pathoman.mskcc.org	Ravichandran *et al.* (2019)

a Truncations in susceptibility genes where LOF is a known mechanism of the disease and harbor variants with a recessive mode of inheritance.

b Truncations when no susceptibility gene list provided.

c Protein length changes due to inframe indels or nonstop variants of genes that harbor variants with a recessive mode of inheritance.

d Protein length changes due to inframe indels or nonstop variants when no susceptibility gene list is provided.

e Peptide change is at the same location as a known benign change.

f Peptide change is known to be benign.

### 3.2 Overall ranking performance

We evaluated each tool’s ability to prioritize disease-causing genes at the top of candidate lists. The radar plot (Fig. 1A) visualizes performance across four key metrics, showing that LIRICAL and Franklin exhibit the largest profiles, indicating superior all-around performance. Ranking accuracy comparisons (Fig. 1B) assessed prioritization performance across multiple thresholds. At the top-1 threshold, Franklin (43.71%) slightly outperformed LIRICAL (39.07%). However, LIRICAL showed stronger performance at the top-5 (62.25% versus 57.62%) and top-10 (68.21% versus 61.59%) thresholds.

### 3.3 Gene retention and distribution across ranking tiers

Gene retention analysis (Fig. 1C) revealed critical differences in filtering strategies. LIRICAL achieved the highest retention rate (75.50%), followed by Franklin (72.19%). Other tools showed excessive filtering, removing the causative gene in most cases: Genebe (72.85% FO), InterVar (78.81% FO), and TAPES (86.75% FO). Rank distribution analysis (Fig. 1D) confirmed the superior early-rank placement by leading tools. LIRICAL and Franklin placed the causative gene as rank 1 in 39% and 43% of cases, respectively. The remaining tools placed the causative gene at rank 1 in less than 8% of cases, with the gene not being found in over 72% of cases.

### 3.4 Precision–recall and balanced performance metrics

**Figure 2.**
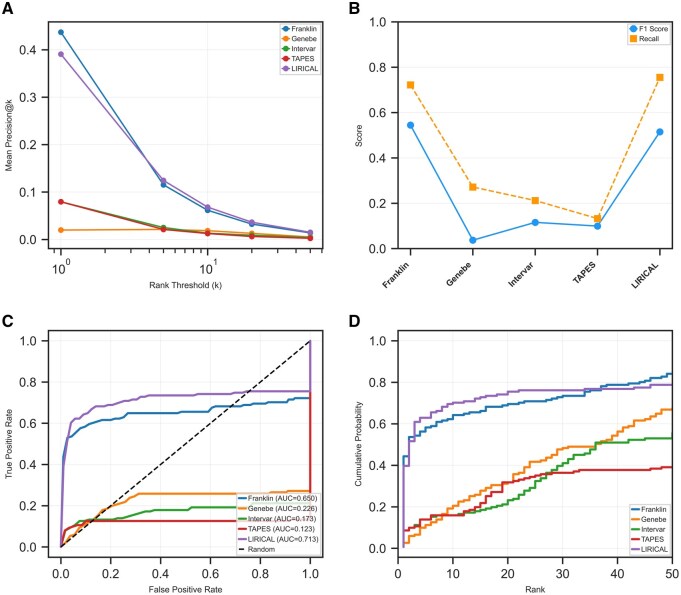
Comparative analysis of precision, recall, and ranking distribution. (A) Mean precision at increasing rank thresholds (*k*). (B) *F*1 score and recall for each tool. (C) Receiver operating characteristic (ROC) curves illustrating the discriminative ability of each tool. (D) Empirical cumulative distribution function (ECDF) plots showing the cumulative probability of finding the causative gene at a given rank.

Precision analysis (Fig. 2A) at various rank thresholds revealed distinct performance patterns. Franklin and LIRICAL achieved the highest precision at rank 1 (43.7% and 39.1%, respectively), and their precision remained highest across all thresholds. *F*1 scores, which balance precision and recall (Fig. 2B), highlighted overall effectiveness. Franklin achieved the highest *F*1 score (0.544), followed closely by LIRICAL (0.515), with other tools scoring 0.116 or lower.

### 3.5 Cumulative ranking performance and discriminative ability

The AUC analysis from the ROC curves (Fig. 2C) provided a unified measure of ranking performance. LIRICAL achieved the highest AUC (0.713), followed by Franklin (0.650), indicating superior discriminative power. Other tools achieved substantially lower AUCs: Genebe (0.226), InterVar (0.173), and TAPES (0.123). The Empirical Cumulative Distribution Functions (ECDFs) of gene ranks (Fig. 2D) further revealed distinct tool behaviors, with LIRICAL and Franklin showing the steepest initial curves, confirming their superior early identification of causative variants.

### 3.6 Statistical validation

**Figure 3.**
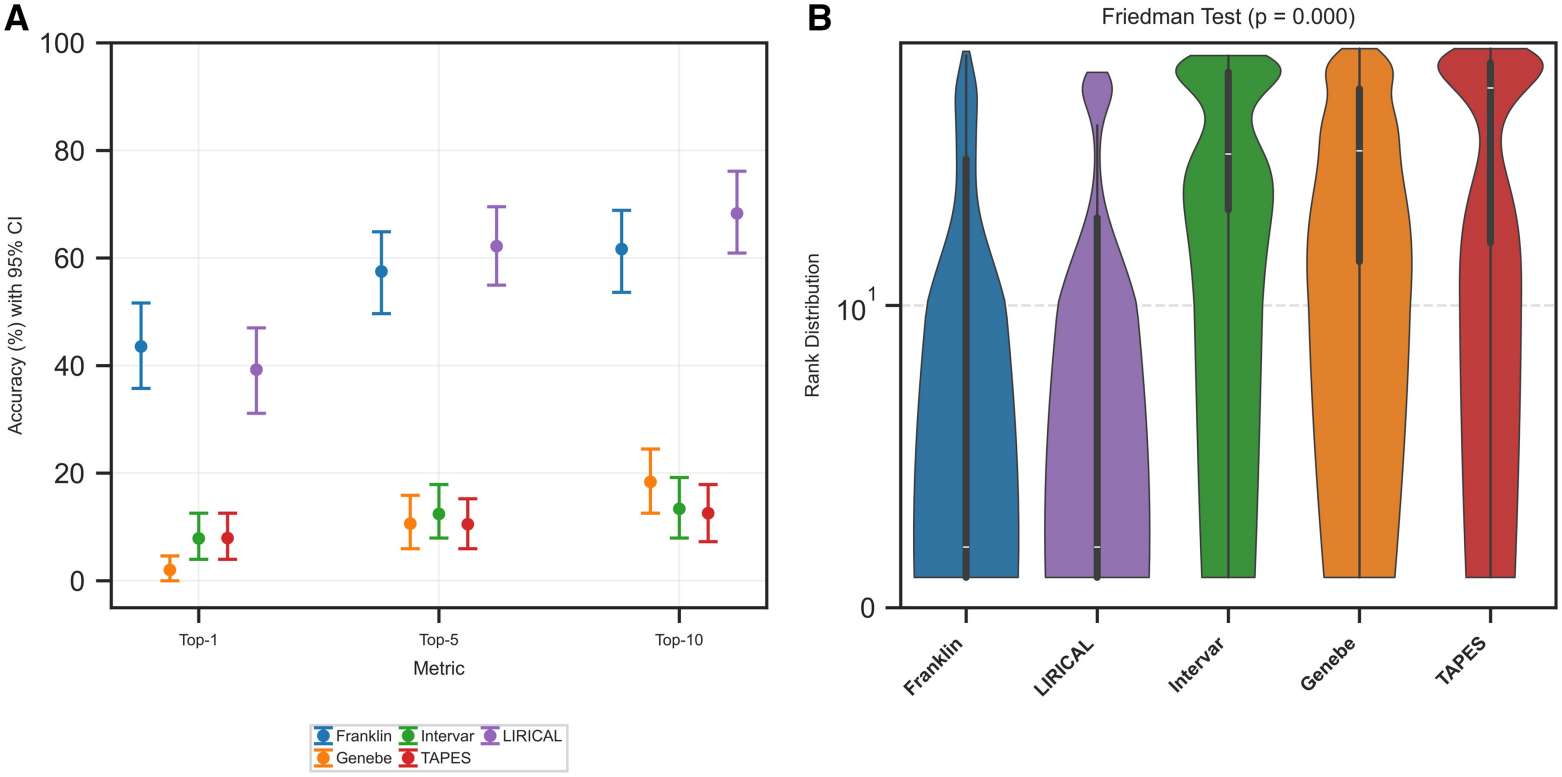
Statistical validation of tool performance. (A) Top-*N* accuracy metrics are shown with 95% bootstrap confidence intervals (CI). (B) The Violin plots show the distinct rank distributions, and as noted in the title, the Friedman test found these differences to be highly significant (*P* = .000).

Bootstrap confidence intervals (*n* = 1000) provided robust estimates of performance reliability (Fig. 3A). Franklin’s top-1 accuracy 95% CI ranged from 35.76% to 51.66%, while LIRICAL's spanned 31.13% to 47.02%. The overlapping intervals suggest comparable top-1 performance.

However, the confidence intervals for these leading tools did not overlap with those of Genebe (0.00%–4.64%), InterVar (3.97%–12.58%), or TAPES (3.97%–12.58%), confirming their significantly lower accuracy. The Friedman test revealed highly significant differences among tools (*χ*^2^ = 159.63, *P* < .0001), confirming that observed performance variations were not due to chance. The violin plots of rank distributions (Fig. 3B) demonstrated distinct clustering, with Franklin and LIRICAL consistently achieving lower median ranks compared to other tools (Table 2).

**Table 2.** Performance comparison of ACMG/AMP-based variant classification tools.

Tool	Top-1 (%)	Top-5 (%)	Top-10 (%)	Top-50 (%)	Retention rate (%)	Median rank	*F*1	AUC
Franklin	43.71	57.62	61.59	69.54	72.19	1.00	0.544	0.650
LIRICAL	39.07	62.25	68.21	75.50	75.50	1.00	0.515	0.713
Genebe	1.99	10.60	18.54	25.83	27.15	8.00	0.037	0.226
TAPES	7.95	10.60	12.58	12.58	13.25	1.00	0.099	0.123
InterVar	7.95	12.58	13.25	21.19	21.19	3.50	0.116	0.173

## 4 Discussion

While high-throughput sequencing has revolutionized genomic diagnostics, the accurate interpretation of variants remains a persistent bottleneck in clinical genomics (Cheng *et al.* 2023). Our study addresses a critical gap in clinical genomics by providing the first comprehensive, direct comparison of guideline-based variant interpretation tools using a unified analytical framework. Since ACMG/AMP guidelines are the foundation for variant classification in most clinical genetic laboratories worldwide, understanding how different tools apply these standards is essential for reliable clinical decision-making. Unlike previous studies that examined tools focused only on phenotype-driven tools (Kelly *et al.* 2022, Yuan *et al.* 2022), or computational prediction algorithms (Chen *et al.* 2020), our research takes a comprehensive approach that mirrors real clinical workflows where multiple types of evidence must be combined according to ACMG/AMP guidelines. This directly addresses a major challenge in precision medicine. While DNA sequencing costs have dropped dramatically, interpreting variants remains a significant obstacle due to the time and expertise required. Our unified comparison represents an important step forward in understanding how these essential clinical tools perform in actual diagnostic settings, filling a major gap in the literature while providing practical guidance for clinical laboratories facing variant interpretation challenges. Among the evaluated tools, Franklin and LIRICAL consistently outperformed others. In contrast, tools such as InterVar, TAPES, and Genebe demonstrated lower performance in prioritizing causal variants, regardless of their adherence to the ACMG/AMP guidelines.

The performance differences observed in our benchmark likely reflect distinct algorithmic approaches implemented by each tool. Franklin employs an artificial intelligence (AI)-based engine that integrates phenotypic and genotypic data within a knowledge base architecture, dynamically adjusting the importance of ACMG/AMP criteria based on phenotypic context (Franklin by Genoox 2025). LIRICAL adopts a probabilistic model using phenotype likelihood ratios, offering robust prioritization even without direct guideline implementation (Robinson *et al.* 2020). Conversely, tools like InterVar, TAPES, and Genebe rely on static rule-based frameworks with no phenotypic integration (Li and Wang 2017, Xavier *et al.* 2019, Stawiński and Płoski 2024). These tools lack sophisticated integration of phenotypic information, which might explain their modest performance. The strong performance of tools that integrate phenotype data supports our hypothesis that this approach significantly enhances variant classification accuracy. This is particularly true for disorders with well-documented genotype-phenotype correlations, where tools can evaluate variants in a more defined context. In contrast, genetically heterogeneous disorders with diverse and often unique phenotypic patterns force tools to rely more heavily on variant-based information when phenotypic context is limited. Our findings align with recent benchmarking studies of phenotype-driven methods. Yuan et al. (2022) demonstrated that approaches combining both HPO terms and VCF files consistently outperformed tools using only phenotypic data or those restricting phenotypic evidence. Their work revealed that LIRICAL and AMELIE, despite different methodological approaches, showed complementary strengths in their rankings (Kelly *et al.* 2022). Similarly, Kelly *et al.* (2022) reported that LIRICAL performed best for retinal disease diagnosis, ranking causal genes within the top-10 candidates for 96% of cases. These results collectively confirm the value of advanced phenotype integration compared to standard implementations that restrict phenotypic evidence to a minor role (Yuan *et al.* 2022).

Our study identifies a key limitation in current variant assessment methods: the inadequate integration of phenotypic data into classification frameworks. The ACMG/AMP guidelines treat phenotypic evidence as merely supporting through PP4 (Richards *et al.* 2015), which limits optimal variant prioritization. Since the release of the ACMG/AMP guidelines, models have been suggested to transform the guidelines into a Bayesian framework (Tavtigian *et al.* 2018). This provides mathematical underpinnings for the recommendations and enables automation of certain aspects of variant pathogenicity assessment (Tavtigian *et al.* 2018). The Clinical Genome Resource (ClinGen) Sequence Variant Interpretation (SVI) Working Group, along with disease-specific Variant Curation Expert Panel (VCEP) Working Groups have also expanded the framework and introduced quantitative approaches covering individual genes, specific diseases, mitochondrial variants, and other well-characterized variants (Oza *et al.* 2018, McCormick *et al.* 2020, Preston *et al.* 2022). The guidance also permits PP4 to reach up to 5 Bayesian points, substantially higher than the 1 point typically assigned to supporting evidence (Biesecker *et al.* 2024). A more effective approach suggested by Basel-Salmon (2024) involves creating categories of disorders based on phenotypic specificity and then assigning monogenic disorders to these categories. This approach acknowledges that when a phenotype or combination of phenotypes is extremely rare, it creates a more distinctive phenotype that should carry greater weight when interpreting variants (Basel-Salmon 2024). Johnson *et al.* (2022) further addressed PP4 limitations through their phenotype diagnostic rate (PDR) approach. When applying “highly predictive phenotype evidence” (PDR >75%), they classified 79.6% of variants as pathogenic/likely pathogenic, compared to only 45.5%–53.5% using approaches that treat phenotypic evidence as merely supporting. This data-driven methodology transforms qualitative assessment into quantifiable metrics, aligning with the ClinGen PP1/PP4 guidance approach (Johnson *et al.* 2022). The practical impact of phenotype-based criteria is demonstrated in recent clinical studies. Shin *et al.* (2025) demonstrated that applying new PP1/PP4 criteria to FBN1 variants of uncertain significance improved reclassification rates to pathogenic/likely pathogenic from 40.3% to 62.5%. This substantial improvement underscores the clinical value of systematic phenotype incorporation in variant classification (Shin *et al.* 2025). In the context of prenatal exome analysis, studies have shown the change in classification of variants when data from post-natal or postmortem evaluations become available (Aarabi *et al.* 2018). Our results, together with others, demonstrate that phenotype-aware classification is not only more accurate, but also more clinically actionable.

Several limitations of our study warrant consideration. Our benchmarking utilizes cases with established diagnoses, which may introduce selection bias toward variants that are more readily detected by current methods. The performance metrics reported here may not generalize to more challenging cases with variants of uncertain significance or in genes with limited disease associations. Additionally, we ran all tools with default parameters to reflect realistic clinical usage scenarios. However, custom parameter optimization might improve the performance of these tools for specific applications.

Based on these findings, robust integration of phenotype data, whether via artificial AI-powered knowledge bases or probabilistic likelihood models, can significantly enhance the variant classification performance. Building on this, future development should explore hybrid frameworks that combine these complementary strengths. In particular, we anticipate a pivotal role for multimodal large language models (MLLMs) that are capable of reasoning over diverse biomedical inputs, including structured phenotypes, clinical narratives, imaging, and genomic annotations (Bhattacharya *et al.* 2024). These LLM-driven systems could facilitate real-time interpretation of variants by synthesizing heterogeneous evidence sources, automating literature curation, and even providing transparent justifications for pathogenicity classifications.

To support clinical translation in the near term, we propose four strategies for consideration, some of which are currently practiced in the context of clinical genetic testing. First, standardization of the phenotypic evaluation should be considered using structured ontologies such as the HPO to ensure data interoperability and reproducibility across laboratories. Second, probabilistic frameworks such as Bayesian inference or likelihood ratio models could be adapted in the analysis pipeline to rigorously integrate phenotypic data with genomic features, enabling more nuanced and quantitative variant interpretation. Third, clinical pipelines should routinely reassess variants of uncertain significance using updated phenotypic evidence and advanced tools such as LIRICAL, enhanced Franklin-like platforms, or future LLM-based curation systems. This is particularly critical in rare disease settings, where reclassification can have significant clinical implications. The rapid evolution of variant classification tools means that newer versions or emerging platforms might demonstrate even better performance characteristics than those observed in our study. Finally, future updates to the variant classification guidelines should consider giving greater weight to phenotypic evidence. In particular, we recommend reconsidering the current supporting-level weight assigned to phenotypic evidence (PP4) within the ACMG/AMP framework and encouraging the development of tools that effectively leverage this critical information. The forthcoming ACMG/AMP guidelines version 4, with its planned replacement of the PP4 category, represents a promising development that should help bridge the gap between current static implementations and the more sophisticated phenotype-aware approaches that our study shows are necessary for optimal variant classification. As precision medicine advances, variant interpretation standards should evolve accordingly to support more accurate genomic diagnostics.

## Supplementary Material

btaf623_Supplementary_Data

## Data Availability

All data and source code required to reproduce the findings of this study are openly available in the Code Ocean repository at https://doi.org/10.24433/CO.6562438.v1.
