# Supplementary material for: Comprehensive evaluation of ACMG/AMP-based variant classification tools
Source: Bioinformatics. 2026 Feb 13;42(2):btaf623. doi: 10.1093/bioinformatics/btaf623 (PMC12916173; doi:10.1093/bioinformatics/btaf623)
Supplement: btaf623_Supplementary_Data [file btaf623_supplementary_data.zip › Supplementary_Materials_for_Online.docx]

**Comprehensive Evaluation of ACMG/AMP-based Variant Classification Tools**


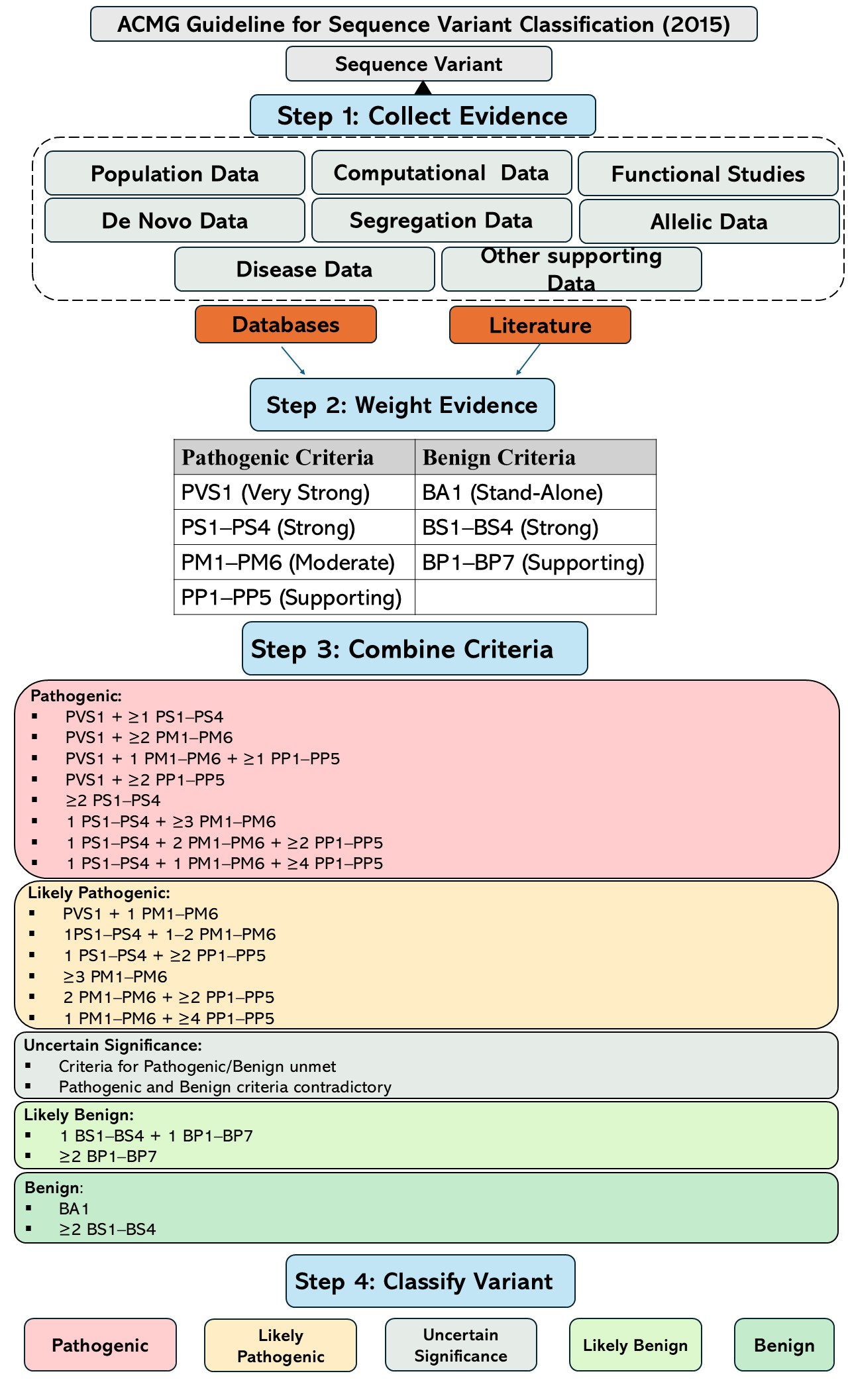
**Supplementary Figures**

**Supplementary Figure 1:** ACMG Guidelines for Sequence Variant Classification (Richards et al., 2015). The process follows four sequential steps: (1) Collection of evidence from various sources (2) Weighting of evidence using specific criteria categorized as pathogenic (PVS, PS, PM, PP) or benign (BA, BS, BP) with varying strengths; (3) Combination of weighted criteria to reach one of five classification outcomes (Pathogenic, Likely Pathogenic, Uncertain Significance, Likely Benign, or Benign) based on specific rule-based combinations; and (4) Final variant classification.

**Supplementary Tables**

**Supplementary Table 1.** ACMG/AMP variant classification framework (Richards et al., 2015) was restructured to highlight key criteria and classification rules. Criteria descriptions are summarized from the original guidelines. See Richards et al. (2015) for complete criteria and caveats.

| **Evidence Level** | **Pathogenic Criteria** | **Benign Criteria** | **Variant Classification Rules** |
| --- | --- | --- | --- |
| Very Strong (PVS1) | Null variant (nonsense, frameshift, canonical ±1 or 2 splice sites, initiation codon, single or multi-exon deletion) in a gene where loss of function is a known mechanism of disease. | Not applicable | Pathogenic:  • PVS1 + ≥1 PS1–PS4  • PVS1 + ≥2 PM1–PM6  • PVS1 + 1 PM1–PM6 + 1 PP1–PP5  • PVS1 + ≥2 PP1–PP5 |
| Strong  (PS1–PS4 / BS1–BS4) | PS1: Same amino acid change as an established pathogenic variant.  PS2: De novo with confirmed paternity and maternity.  PS3: Well-established functional studies support damaging effect.  PS4: Significantly increased prevalence in affected individuals vs. controls. | BS1: Allele frequency greater than expected for disorder.  BS2: Observed in healthy adult for fully penetrant early-onset disorder.  BS3: Functional studies show no damaging effect.  BS4: Lack of segregation in affected family members. | Pathogenic:  • ≥2 PS1–PS4  • 1 PS1–PS4 + ≥3 PM1–PM6  • 1 PS1–PS4 + 2 PM1–PM6 + ≥2 PP1–PP5  • 1 PS1–PS4 + 1 PM1–PM6 + ≥4 PP1–PP5 |
| Moderate  (PM1–PM6) | PM1: Located in a mutational hot spot or critical functional domain.  PM2: Absent from controls in large population databases.  PM3: For recessive disorders, detected in trans with a pathogenic variant.  PM4: Protein length changes due to in-frame deletions/insertions or stop-loss.  PM5: Novel missense at a residue where different pathogenic missense seen before.  PM6: Assumed de novo without confirmed paternity and maternity. | Not applicable | Likely Pathogenic:  • PVS1 + 1 PM1–PM6  • 1 PS1–PS4 + 1–2 PM1–PM6  • 1 PS1–PS4 + ≥2 PP1–PP5  • ≥3 PM1–PM6  • 2 PM1–PM6 + ≥2 PP1–PP5  • 1 PM1–PM6 + ≥4 PP1–PP5 |
| Supporting  (PP1–PP5 / BP1–BP7) | PP1: Co-segregation with disease in multiple affected family members.  PP2: Missense in gene with low benign missense rate.  PP3: Multiple lines of computational evidence support deleterious effect.  PP4: Patient's phenotype or family history highly specific for gene.  PP5: Reputable source reports as pathogenic. | BP1: Missense in gene where only truncating variants cause disease.  BP2: Observed in trans with pathogenic variant for dominant disorder, or in cis with pathogenic variant.  BP3: In-frame deletions/insertions in repetitive region without known function.  BP4: Multiple lines of computational evidence suggest no impact.  BP5: Variant found in case with alternate molecular basis.  BP6: Reputable source reports as benign.  BP7: Synonymous variant with no predicted splice impact and not conserved. | Likely Benign:  • 1 BS1–BS4 + 1 BP1–BP7  • ≥2 BP1–BP7 |
| Stand-alone  (BA1) | Not applicable | BA1: Allele frequency >5% in large population databases. | Benign:  • BA1  • ≥2 BS1–BS4 |
| - | - | - | Uncertain Significance:  • Criteria for Pathogenic/Benign not met  • Pathogenic and Benign criteria are contradictory |

**Supplementary Table 2.** Compilation of datasets employed in the research

| **Mendelian Disorders Type** | **NO.** | **Sources** |
| --- | --- | --- |
| Osteogenesis imperfecta | 33 | Laboratory of Chulalongkorn |
| Congenital tooth anomalies | 62 | Laboratory of Chulalongkorn |
| Hearing loss | 14 | Laboratory of Chulalongkorn |
| Retinitis pigmentosa | 21 | Salmaninejad et al.[42,43] |
| Ameloblastoma | 21 | Laboratory of Chulalongkorn |

**Search Strategy:**

(((((((((((((((((Automatized) **OR** (Computerized)) **OR** (Automated)) **OR** (Machine learning)) **OR** (Mechanized)) **OR** (Digitalized)) **OR** (Autonomous)) **OR** (Calculator)) **OR** (calculation)) OR ("artificial intelligence")) **OR** (tool)) **OR** (software)) **OR** (command-line)) **OR** (website)) **OR** (interface)) **OR** (Algorithm)) **OR** (modelling)) **OR** (platform) **OR** (System**) OR** (model)) **AND** (("American College of Medical Genetics and Genomics") **OR** (ACMG)) OR (ACMG/AMP)

Search conducted til: 10/5/2024

Date restriction of: nil.

**Exclusion criteria:**

- Publications focusing only on variant prioritization and not full according to ACMG guidelines
- Publications describing purely expert-based/manual variant classifications without automation/computational aspects
- Publications focusing on variant classifications for non-human species
- Publications in languages other than English
- Publications prior to 2015 (to exclude studies from before establishment of standardized ACMG guidelines)
- Editorials, comments, letters to editors,
- **Inclusion criteria:**
- Publications from 2015 onwards (to focus on most recent methods after establishment of ACMG guidelines)
- Publications that developed automated variant classification methods based on ACMG/AMP guidelines

**Search Results:**

- PubMed results: 537

**Calculations:**

n(Total) = 537

Key articles: 21
