# Supplementary material for: Comprehensive evaluation of ACMG/AMP-based variant classification tools
Source: Bioinformatics. 2026 Feb 13;42(2):btaf623. doi: 10.1093/bioinformatics/btaf623 (PMC12916173; doi:10.1093/bioinformatics/btaf623)
Supplement: btaf623_Supplementary_Data [file btaf623_supplementary_data.zip › 01-Jan-2026_083459_Supplementary_Materials_for_Online.docx]

**Comprehensive Evaluation of ACMG/AMP-based Variant Classification Tools**

**Supplementary Figures**

**
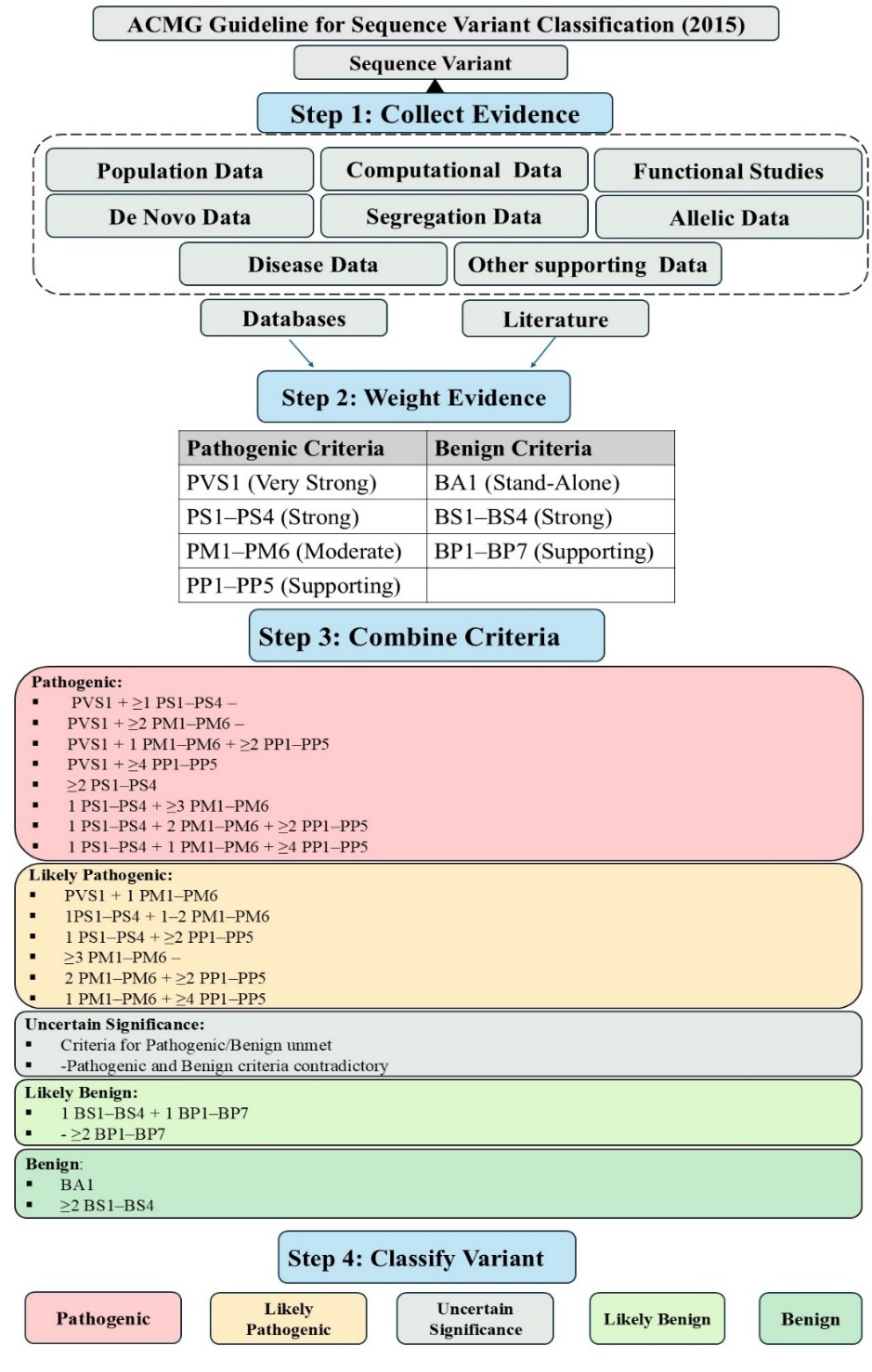
**

**Supplementary Figure 1:** ACMG Guidelines for Sequence Variant Classification (Richards et al., 2015). The process follows four sequential steps: (1) Collection of evidence from various sources (2) Weighting of evidence using specific criteria categorized as pathogenic (PVS, PS, PM, PP) or benign (BA, BS, BP) with varying strengths; (3) Combination of weighted criteria to reach one of five classification outcomes (Pathogenic, Likely Pathogenic, Uncertain Significance, Likely Benign, or Benign) based on specific rule-based combinations; and (4) Final variant classification.

**Supplementary Tables**

**Supplementary Table 1.** ACMG/AMP variant classification framework (Richards et al., 2015) was restructured to highlight key criteria and classification rules.

| **Evidence Level** | **Pathogenic Criteria** | **Benign Criteria** | **Variant Classification Rules** |
| --- | --- | --- | --- |
| Very Strong (PVS1) | Null variant in a gene where loss of function is a known mechanism of disease. | Not applicable | Pathogenic: 1 Very Strong AND (≥1 Strong OR ≥2 Moderate OR 1 Moderate and 1 supporting OR ≥2 Supporting) |
| Strong (PS1-PS4) | 1. Same amino acid change as a pathogenic variant. 2. De novo with confirmed paternity and maternity. 3. Functional studies support damaging effects. 4. Increased prevalence in affected individuals. | 1. Allele frequency greater than expected for the disorder. 2. Observed in healthy adults for a fully penetrant disorder at an early age. 3. Functional studies show no damaging effect. 4. Lack of segregation in affected families. | Likely Pathogenic: 1 Strong AND (1-2 Moderate OR ≥2 Supporting) |
| Moderate (PM1-PM6) | 1. Located in a hot spot or critical domain. 2. Absent from controls in large databases. 3. For recessive disorders, detected in trans with a pathogenic variant. 4. Protein length changes due to in-frame deletions/insertions or stop-loss variants. 5. Novel missense change at a known pathogenic residue. 6. Assumed de novo without confirmed paternity and maternity. | Not applicable | Likely Pathogenic: ≥3 Moderate OR 2 Moderate AND ≥2 Supporting OR 1 Moderate AND ≥4 Supporting |
| Supporting (PP1-PP5) | 1. Co-segregation with disease in the family. 2. Missense variant in a gene with low benign missense rate. 3. Multiple lines of computational evidence support deleterious effects. 4. Specific phenotype or family history. 5. Reputable source reports as pathogenic. | 1. Missense variant in a gene known for truncating variants. 2. Observed in trans or cis with a pathogenic variant. 3. In-frame deletions/insertions in nonfunctional regions. 4. Computational evidence suggests no impact. 5. Variant found in cases with alternate disease cause. 6. Reputable source reports as benign. 7. Synonymous variant not impacting splice sites and not conserved. | Likely Benign: ≥2 Supporting |
| Stand-alone | Not applicable | Allele frequency >5% in large databases. | Benign: 1 Stand-alone OR ≥2 Strong |
| - | - | - | Uncertain Significance: Other criteria not met OR criteria for benign and pathogenic are contradictory. |

**Supplementary Table 2.** Compilation of datasets employed in the research

| **Mendelian Disorders Type** | **NO.** | **Sources** |
| --- | --- | --- |
| Osteogenesis imperfecta | 33 | Laboratory of Chulalongkorn |
| Congenital tooth anomalies | 62 | Laboratory of Chulalongkorn |
| Hearing loss | 14 | Laboratory of Chulalongkorn |
| Retinitis pigmentosa | 21 | Salmaninejad et al.[42,43] |
| Ameloblastoma | 21 | Laboratory of Chulalongkorn |

**Search Strategy:**

(((((((((((((((((Automatized) **OR** (Computerized)) **OR** (Automated)) **OR** (Machine learning)) **OR** (Mechanized)) **OR** (Digitalized)) **OR** (Autonomous)) **OR** (Calculator)) **OR** (calculation)) OR ("artificial intelligence")) **OR** (tool)) **OR** (software)) **OR** (command-line)) **OR** (website)) **OR** (interface)) **OR** (Algorithm)) **OR** (modelling)) **OR** (platform) **OR** (System**) AND** (("American College of Medical Genetics and Genomics") **OR** (ACMG)) OR (ACMG/AMP)

Search conducted til: 10/5/2024

Date restriction of: nil.

**Exclusion criteria:**

- Publications focusing only on variant prioritization and not full according to ACMG guidelines
- Publications describing purely expert-based/manual variant classifications without automation/computational aspects
- Publications focusing on variant classifications for non-human species
- Publications in languages other than English
- Publications prior to 2015 (to exclude studies from before establishment of standardized ACMG guidelines)
- Editorials, comments, letters to editors,
- **Inclusion criteria:**
- Publications from 2015 onwards (to focus on most recent methods after establishment of ACMG guidelines)
- Publications that developed automated variant classification methods based on ACMG/AMP guidelines

**Search Results:**

- PubMed results: 537

**Calculations:**

n(Total) = 537

Key articles: 21
